# Comparison of carina-based versus bony anatomy-based registration for setup verification in esophageal cancer radiotherapy

**DOI:** 10.1186/s13014-018-0986-1

**Published:** 2018-03-21

**Authors:** Mélanie Machiels, Peng Jin, Christianne H. van Gurp, Jeanin E. van Hooft, Tanja Alderliesten, Maarten C. C. M. Hulshof

**Affiliations:** 1 Department of Radiation Oncology, Academic Medical Center, University of Amsterdam, Meibergdreef 9, 1105 AZ Amsterdam, the Netherlands; 2 Department of Gastroenterology and Hepatology, Academic Medical Center, University of Amsterdam, Amsterdam, the Netherlands

**Keywords:** Esophageal cancer, Radiotherapy, Fiducial markers, Setup verification, Carina-based registration, Image-guided radiotherapy

## Abstract

**Background:**

To investigate the feasibility and geometric accuracy of carina-based registration for CBCT-guided setup verification in esophageal cancer IGRT, compared with current practice bony anatomy-based registration.

**Methods:**

Included were 24 esophageal cancer patients with 65 implanted fiducial markers, visible on planning CTs and follow-up CBCTs. All available CBCT scans (*n* = 236) were rigidly registered to the planning CT with respect to the bony anatomy and the carina. Target coverage was visually inspected and marker position variation was quantified relative to both registration approaches; the variation of systematic (Σ) and random errors (σ) was estimated.

**Results:**

Automatic carina-based registration was feasible in 94.9% of the CBCT scans, with an adequate target coverage in 91.1% compared to 100% after bony anatomy-based registration. Overall, Σ (σ) in the LR/CC/AP direction was 2.9(2.4)/4.1(2.4)/2.2(1.8) mm using the bony anatomy registration compared to 3.3(3.0)/3.6(2.6)/3.9(3.1) mm for the carina. Mid-thoracic placed markers showed a non-significant but smaller Σ in CC and AP direction when using the carina-based registration.

**Conclusions:**

Compared with a bony anatomy-based registration, carina-based registration for esophageal cancer IGRT results in inadequate target coverage in 8.9% of cases. Furthermore, large Σ and σ, requiring larger anisotropic margins, were seen after carina-based registration. Only for tumors entirely confined to the mid-thoracic region the carina-based registration might be slightly favorable.

**Electronic supplementary material:**

The online version of this article (10.1186/s13014-018-0986-1) contains supplementary material, which is available to authorized users.

## Background

Treatment policy for esophageal cancer has substantially changed in the last decade, with radiotherapy playing an increasingly important role in both neo-adjuvant and definitive treatment [1, 2]. Esophageal radiotherapy encompasses considerable geometrical uncertainties due to setup errors, position variation of the esophageal target volume, and organ motion. Image-guided radiotherapy (IGRT) was developed to reduce these geometrical uncertainties by acquiring images of the patient’s anatomy directly prior to treatment and comparing these with the anatomy during treatment planning, by rigidly registering the 3-dimensional (3D) planning computed tomography (pCT) with the kilo−/megavoltage (kV/MV) cone-beam CT (CBCT) on the bony anatomy (i.e., vertebrae) [3]. Target volume misalignments are subsequently assessed and setup corrections are made (generally) through a couch shift.

Ideally for esophageal tumors, a tumor-based registration is used. However, with the limited soft-tissue contrast in CT, and especially CBCT with its even lower contrast resolution, the tumor cannot be discriminated reliably from its surrounding tissues. For standard clinical use, the well-visible bony anatomy (i.e., vertebrae) is currently used as a tumor surrogate in the CBCT-guided setup verification procedure. Intra/para-tumoral fiducial markers have been investigated as a tumor surrogate for setup verification. Unfortunately, a recent study at our department demonstrated that rigid marker-based registration is unfeasible due to tissue deformation [4].

Large intra- and interfractional tumor position variation is observed at the daily treatment fractions when a setup according to bony anatomy in esophageal cancer is used [4–7]. To account for such geometrical uncertainties in the absence of adequate correction strategies, the use of generous safety margins is necessary. Nevertheless, these substantial margins expose nearby organs to increased radiation doses, which might increase toxicity and impede the use of dose escalation [2, 8, 9].

With its close proximity to the esophagus and the mediastinal lymph nodes, clear visibility on CT and CBCT, and its ability to move during respiration as opposed to the rigid and stable vertebrae, the carina might be superior to the vertebrae as a tumor surrogate for setup verification and might reduce interfractional position variation, as was demonstrated in lung tumors [10, 11].

The aim of the present study was to investigate the feasibility and assess the target coverage and geometric accuracy of a carina-based registration for CBCT-guided setup verification in esophageal cancer IGRT, compared with current practice bony anatomy-based registration. Moreover, the use of fiducial markers allowed to quantify interfractional position variation in all directions, including the otherwise hard to determine craniocaudal (CC) direction, and made it possible to compare different subgroups based on their locations in the esophagus.

## Methods

### Patient and marker characteristics

From March 2013 to May 2014, 30 esophageal cancer patients were consecutively included in this retrospective study. This patient population is identical to the one used in a previous study examining the feasibility of a marker-based registration [4]. The population consisted of 24 males and 6 females, aged 45–84 (average 66) years. For each patient, at least 2 fiducial markers were placed at esophageal tumor borders under endoscopy/endoscopic ultrasonography (EUS) guidance. Due to tumor characteristics (e.g., stenosis, stricture, ...) placement of a marker at the tumor border was not always feasible. In that case, a marker was placed as close as possible and the distance to the true border was assessed by measuring the distance between the implanted marker and the border under fluoroscopy, placing the tip of the scope at the tumor border. This procedure was earlier approved by our institution’s medical ethics committee and all patients had given written informed consent [4, 12]. Patient details are listed in Table 1, with patient numbering consistent with the previous in-house study on interfractional tumor position variation [4]. Three types of fiducial markers were implanted: a solid gold marker (Cook Medical, Limerick, Ireland), a flexible coil-shaped gold marker (Visicoil; IBA Dosimetry, Bartlett, TN, USA), or a radiopaque hydrogel marker (TraceIt; Augmenix, Waltham, MA, USA).

**Table 1:** Patient, tumor and marker characteristics

Patient	Age (yr)	Sex	Tumor length (cm)	TNM	Histology	Tumor location	Marker type	No. of CBCTs	No. of markers		
									At placement	Visible in pCT	Visible in CBCTs
1	56	M	5	T3N1M0	AD	distal	SM	7	2	1	1
2	79	M	8	T3N1M0	AD	distal	SM	7	3	2	1
3	62	M	8	T3N1M0	AD	distal	VM	7	3	3	1
4	61	M	4	T3N1M0	PDC	distal	SM	7	3	3	3 → 2
5^a^	73	M	3	T2N1M0	SCC	distal	HG	8^a^	0	0	0^a^
6	57	V	3	T2N0M0	SCC	distal	SM	8	4	4	4
7	63	M	2	T3N2M0	AD	distal	SM	8	4	4	4
8^a^	83	M	8	T2N2M0	AD	distal	VM	28^a^	5	5	0^a^
9	65	V	4	T2N0M0	SCC	mid	VM	8	4	3	3
10^a^	57	M	5	T2N0M0	AD	distal	VM	7^a^	3	2	0^a^
11	64	M	5	T3N2M0	SCC	distal	SM	12	3	2	2
12	70	M	13	T2N1M0	SCC	proximal	SM	8	3	3	3
13	67	V	5	T3N2M0	SCC	mid	VM	23	4	4	4
14	73	M	4	T2N0M0	SCC	proximal	VM	25	3	3	1
15^a^	71	M	6	T4aN2M0	SCC	distal	HG	8^a^	6	5	0^a^
16	84	M	5	T3N1M0	AD	distal	HG	8	3	3	3 → 1
17	61	M	7	T3N1M0	AD	distal	VM	8	3	3	3
18^a^	69	M	7	T3N1M0	AD	distal	HG	23^a^	3	3	0^a^
19	45	M	6	T2N1M0	AD	distal	SM	7	3	3	3 → 2
20	63	M	3	T2N0M0	AD	distal	SM	11	3	2	2
21	79	V	6	T3N2M0	AD	distal	VM	8	4	4	3
22	59	V	10	T3N2M0	AD	distal	VM	9	4	4	4
23	61	M	6	T3N2M0	AD	distal	VM	12	3	3	3
24	69	M	5	T3N0M0	AD	distal	VM	12	4	4	2
25	69	M	8	T2N1MO	AD	distal	VM	8	4	4	4
26^a^	76	M	3	T2N1M0	SCC	distal	HG	8^a^	3	2	0^a^
27	67	V	4.5	T1N0M0	SCC	proximal	SM	8	3	3	3
28	65	M	4	T3N2M0	SCC	mid	VM	9	5	4	4
29	75	M	5	T3N1M0	SCC	distal	VM	9	3	2	2
30	51	M	17	T3N2M0	AD	prox-mid-dist	SM	7	3	3	2
Total								318 → 236	101	91	65 → 61

Patient numbering is consistent with that in Jin et al. [4]

*Abbreviations: AD* adenocarcinoma, *SCC* squamous cell carcinoma, *PDC* poorly differentiated carcinoma, *SM* solid, rigid golden marker, *VM* Visicoil marker, *HG* radiopaque hydrogel, *pCT* planning computed tomography, *CBCT* cone-beam computed tomography

^a^Marker not visible on CBCT, subsequent exclusion of patient from data analysis

**Fig. 1:**
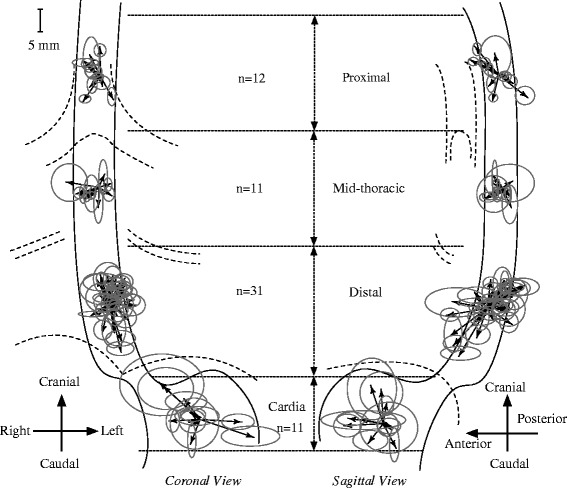
Illustration of the systematic error (SE, the length of the arrow) and the SD of random errors (sdRE, the length of semi-major/minor axis of the ellipse) of each marker position relative to the carina, projected onto the coronal (left) and sagittal (right) views of the esophagus. Note: the amplitudes of the errors are not scaled to the esophagus

All patients with lost or invisible fiducial markers on CT or CBCT were excluded from the analysis (Table 1), resulting in the inclusion of 24 patients with a total of 65 clearly visible fiducial markers on CBCT and CT [4]. Fiducial markers were classified according to the American Joint Committee on Cancer manual into four subgroups based on their locations in the esophagus: proximal (*n* = 12), mid-thoracic (*n* = 11), distal (*n* = 31), and cardia (*n* = 11), respectively [13] (Fig. 1).

### Image acquisition and target delineation

For treatment planning, a 3D pCT scan was acquired within 0–5 days (average: 1 day) after fiducial marker placement; patients were under free-breathing conditions in supine position, with arms raised above their heads. All CTs (axial slice thickness, 2.5/3.0 mm; in-plane pixel size, 1.0, 1.2, or 1.3 mm depending on the field of view of the scan) had a scan area from the bottom edge of the mandible to the lower border of the kidneys.

A gross tumor volume (GTV) was delineated by the radiation oncologist on the pCT based on fiducial marker positions using all available resources, including data from PET/CT fusion scans, EUS reports, and diagnostic CT images. The GTV was expanded to the clinical target volume (CTV) by extending the radiation coverage 3.5 cm in CC directions, or 2.0 cm into the gastric mucosa if there was cardia involvement. In radial direction, all regional lymph nodes and peri-esophageal fatty tissue were incorporated. The planning target volume (PTV) was generated by using a uniform 1.0 cm expansion beyond the borders of the CTV, as has been introduced in the CROSS trial and is currently common clinical practice [2].

As consistent with the extended no action level protocol (eNAL) [14], per patient a total of 7–8 CBCT scans (Elekta Synergy System; Elekta Ltd., Crawley, UK) were acquired before irradiation after initial laser alignment for setup verification. This consisted of a daily CBCT acquisition for the first 4 consecutive fractions, followed by once-weekly acquisitions over the rest of the treatment course. For the fractions without CBCT, patients were positioned based on the average setup error calculated using the available CBCTs. More CBCT scans were acquired when the results of eNAL exceeded tolerance (e.g., patients 13 and 14). In total, 236 CBCT scans of the 24 patients with clearly visible fiducial markers were included in the analysis (Table 1).

### Image registration and target coverage

**Fig. 2:**
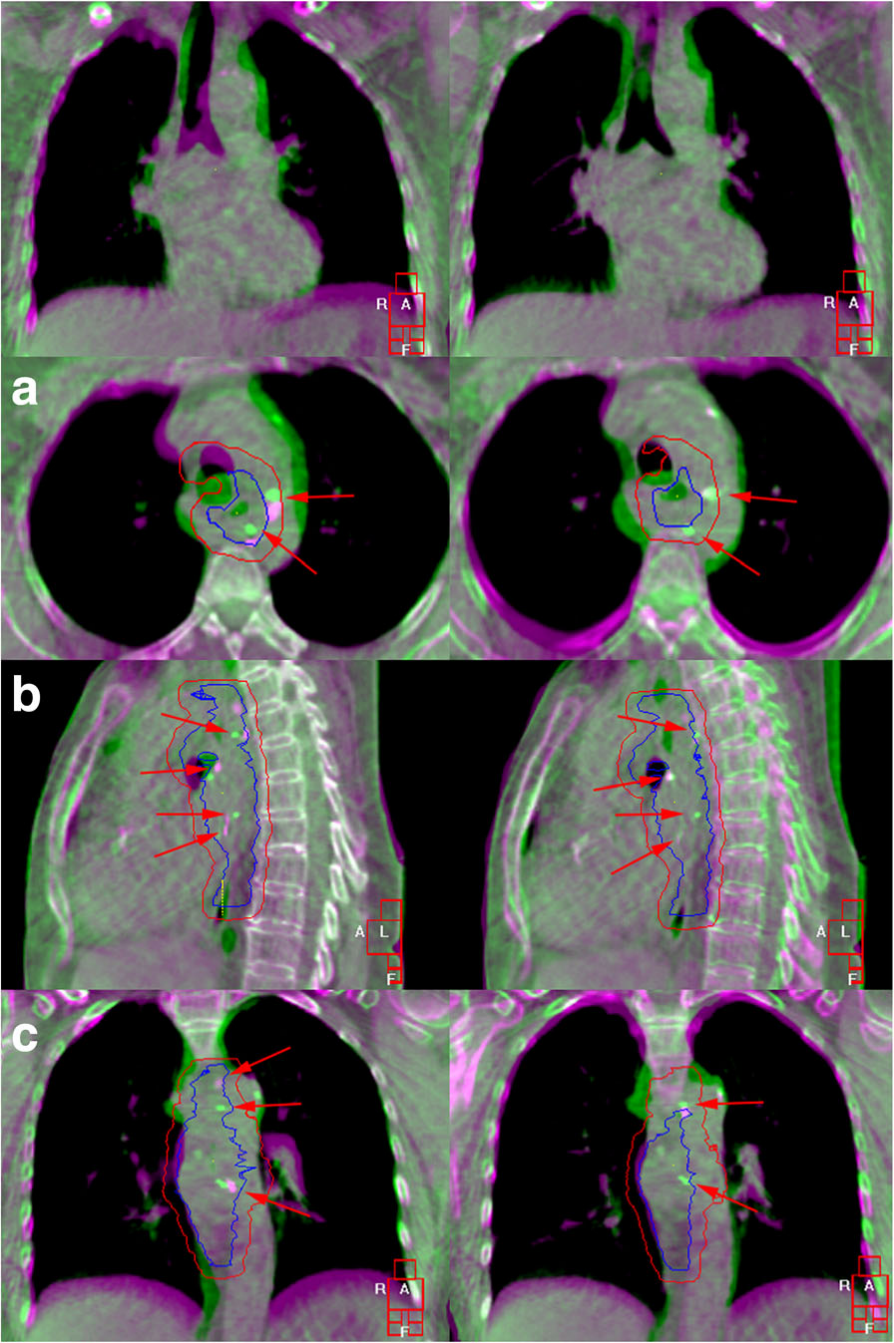
Overlay of the pCT (purple) and CBCT (green) in XVI for patient 9. Arrows indicate fiducial markers, registered on the bony anatomy (left column) and the carina (right column). In axial (**a**), sagittal (**b**), and coronal view (**c**). First row = general carina image in coronal view. A = anterior, R = right, L = left, and F = inferior. Red contour = PTV, blue contour = CTV

For each patient, all available CBCTs were rigidly registered to the pCT using 3D translations and rotations. All registrations were done by two experienced radiation therapists within Elekta X-ray volume imaging (XVI) software (version 4.5; Elekta Oncology Systems), based on the bony anatomy (i.e., vertebrae) and the carina, respectively (Fig. 2). For the bony anatomy registration, a clipbox (i.e., cubic region of interest) partly placed around the cervical/thoracic vertebrae and the Chamfer-matching algorithm was used [15].

Following the bony anatomy registration a carina-based registration was done, using a so-called mask (i.e., shaped region of interest) combined with a gray-value matching algorithm [15]. All registrations were visually checked and manually adjusted when necessary. When the carina-based registration was difficult due to, e.g., deformation of the carina, a manual registration was performed, which aimed to achieve the best visual 3D overlap.

For both the bony anatomy-based registration and the carina-based registration, we subsequently investigated the target coverage as follows. The registered CBCT was visualized together with the PTV delineation derived from the pCT and it was assessed whether the PTV encompassed the CTV as visible in the registered CBCT, this target coverage was scored binary (e.g., adequate vs. inadequate = geographic miss), without taking clinical relevance into account. The fiducial markers were used as an aid in this process.

### Geometric accuracy

To determine the most opportune registration approach for esophageal cancer setup verification, we quantified the systematic error (SE) and the standard deviation (SD) of the random error (sdRE) of the esophageal tumor position, as defined by the fiducial marker positions, relative to the bony anatomy and relative to the carina. In retrospect, for all patients we rigidly registered each CBCT to the pCT based on the bony anatomy or the carina, using XVI software (as described above). Then, per registration approach, for each registered CBCT we calculated the position variation of each fiducial marker relative to its corresponding fiducial marker position in the pCT, in the left-right (LR), CC, and anterior-posterior (AP) direction. Since the fiducial marker position variation was found to be dependent on direction and location in the previous study (attributable to the elongated shape of the esophagus) fiducial marker position variations were analyzed for the whole marker group and for the four marker subgroups based on their locations in the esophagus [4].

The mean and SD of the interfractional marker position variation were subsequently calculated, which are estimates of the SE and the sdRE for individual markers, respectively. Further, for the whole marker group and the four marker subgroups we estimated the group mean (M, the mean of SEs), the SD of SEs (Σ), and the root mean square of sdRE (σ) using a bony anatomy-based registration or a carina-based registration in all three directions (LR, CC, and AP), respectively [16]. Afterwards, for both approaches separately, the margins required to compensate solely for the derived interfractional position errors were calculated pro forma, based on the margin recipe 2.5Σ + 0.7σ [16].

### Statistical analysis

Patient characteristics, registration outcomes (i.e., setup errors), and target coverage were summarized using descriptive statistics. The absolute SE of the interfractional marker position variation in each direction and each subgroup was compared between the two registration approaches by applying a Wilcoxon signed-rank test. Results with *p* < 0.05 were considered to be significant. All statistical analyses were performed using the R software package (version 3.0.2, R Foundation for Statistical Computing, Vienna, Austria).

## Results

### Image registration and target coverage

Automatic carina-based registration was feasible in 224 of the 236 CBCT scans (94.9%), whereas 6 of the 12 CBCTs (50%) of patient 11, and 6 of the 23 CBCTs (26.1%) of patient 13, required a manual registration due to a deformation of the carina. In comparison, an automatic bony anatomy-based registration was feasible in all cases without need for manual adjustments.

**Fig. 3:**
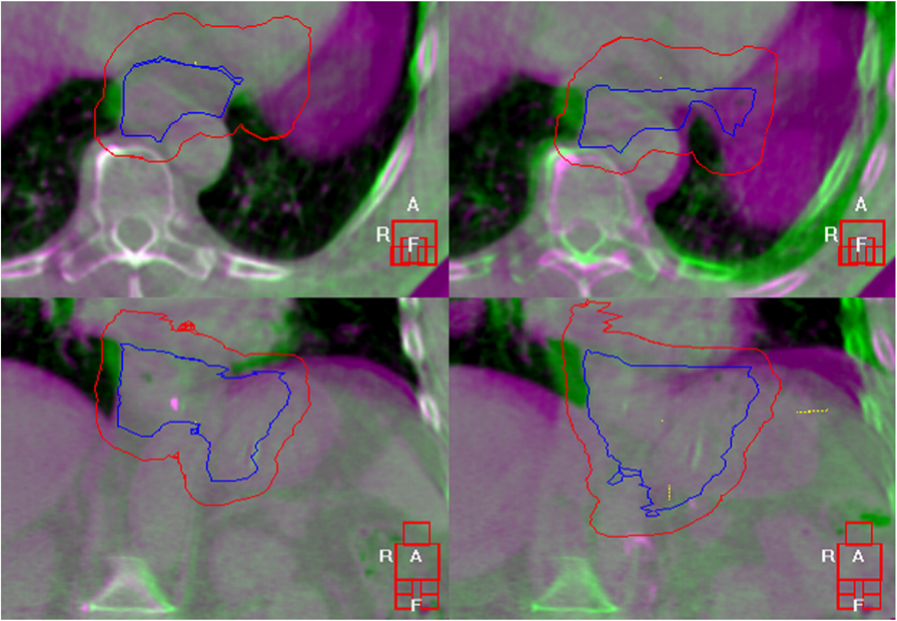
Example of inadequate target coverage after registration. Overlay of the pCT (purple) and CBCT (green) in XVI for patient 20, registered on the bony anatomy (left column) and the carina (right column). In axial (first row) and coronal view (second row). A = anterior, R = right, and F = inferior. Red contour = PTV, blue contour = CTV

After carina-based registration, the PTV encompassed the target volume in 91.1% versus 100% after bony anatomy-based registration. Most geographic misses were seen in patient 11, in which 4 of 12 (33.3%) registrations led to inadequate target coverage, and in patient 20: showing inadequate target coverage in 4 of 11 (36.3%) registrations (Fig. 3).

### Geometric accuracy

**Fig. 4:**
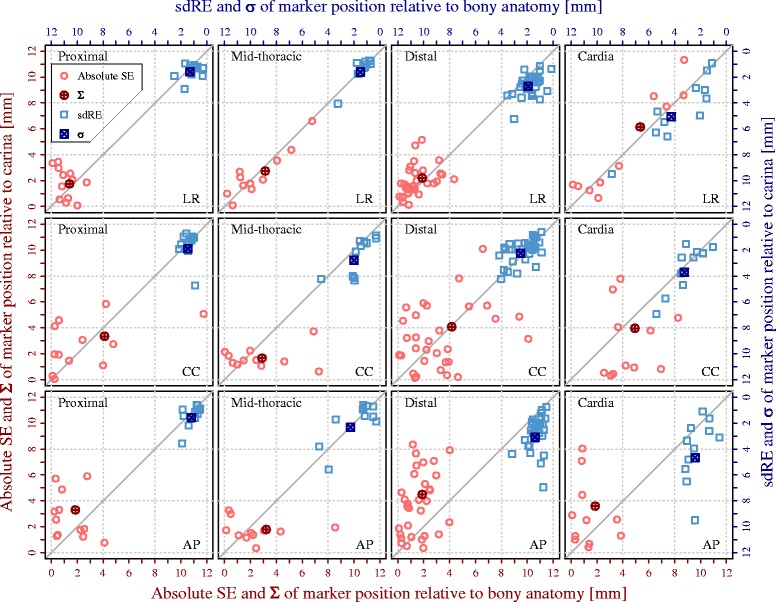
Absolute systematic error (SE) and standard deviation (SD) of random errors (sdRE) of the marker position relative to the bony anatomy and carina. The SD of SEs (Σ) and the root mean square of sdREs (σ) are also depicted

Large SEs and sdREs were found using both registration approaches (Fig. 4 and Additional file 1: Figure S1). For each of the 65 markers, Fig. 4 illustrates the absolute SE and the sdRE, respectively, relative to the bony anatomy and to the carina, for all three directions and all marker subgroups. No advantage of a carina-based registration was seen, with (for most regions/directions) an even larger SE and sdRE using this carina-based registration. Only for markers located directly at the carina we found a smaller SE in all three directions, i.e. CC direction (Σ_carina_: 1.7 mm vs. Σ_bony_: 2.9 mm), LR direction (Σ_carina_: 2.7 mm vs. Σ_bony_: 3.1 mm), and AP direction (Σ_carina_: 1.8 mm vs. Σ_bony_: 3.2 mm); this difference was not significant, but sample size was small.

The M, Σ, σ, and the estimated margins in all directions for the four marker subgroups, and the entire marker group, are presented in Table 2. Largest SEs and consequential Σ were seen predominantly in the CC direction (overall Σ_carina_: 3.6 mm vs. overall Σ_bony_: 4.1 mm) and in the LR direction in the cardia (e.g., LR Σ_carina_: 6.1 mm vs. LR Σ_bony_: 5.4 mm). Only for mid-thoracic esophageal tumors a smaller Σ and margin reduction was seen in three directions, using a carina-based registration compared to a bony anatomy-based registration in CC direction (margin: 6.1 mm vs. 8.6 mm), LR direction (margin: 8.0 mm vs. 8.9 mm) and in AP direction (margin: 6.1 mm vs. 9.6 mm); however, this difference was not significant (*p* = 0.36, *p* = 0.64 and *p* = 0.46, respectively).

**Table 2:** The group mean (M), SD of systematic errors (Σ), root mean square of SDs of random errors (σ), and the estimated margin required to compensate for the interfractional tumor position variation in each orthogonal direction for the four subgroups of markers and all markers (Overall)

		LR (mm)		CC (mm)		AP (mm)	
		Bony anatomy	Carina	Bony anatomy	Carina	Bony anatomy	Carina
Proximal (*n* = 12)	M	0.1	−1.3	−1.2	0.5	− 0.7	1.0
	Σ	1.5	1.7	4.1	3.3	1.9	3.3
	σ	1.3	1.6	1.5	1.9	1.2	1.6
	2.5 Σ + 0.7σ	4.4	5.5	11.3	9.7	5.5	9.3
Mid-thoracic (*n* = 11)	M	−1.1	−1.5	−2.3	−1.0	1.2	0.8
	Σ	3.1	2.7	2.9	1.7	3.2	1.8
	σ	1.5	1.6	2.0	2.8	2.3	2.3
	2.5 Σ + 0.7σ	8.9	8.0	8.6	6.1	9.6	6.1
Distal (*n* = 31)	M	0.4	0.8	−0.8	0.5	0.5	−0.8
	Σ	1.9	2.2	4.2	4.1	1.9	4.5
	σ	1.9	2.7	2.5	2.2	1.4	3.1
	2.5 Σ + 0.7σ	6.1	7.4	12.1	11.7	5.7	13.4
Cardia (*n* = 11)	M	−1.1	−1.2	−1.2	0.9	−0.1	1.7
	Σ	5.4	6.1	4.9	4.0	1.9	3.6
	σ	4.3	5.1	3.2	3.7	2.4	4.7
	2.5 Σ + 0.7σ	16.4	18.9	14.6	12.5	6.4	12.2
Overall (*n* = 65)	M	−0.2	−0.3	−1.2	0.3	0.3	0.2
	Σ	2.9	3.3	4.1	3.6	2.2	3.9
	σ	2.4	3.0	2.4	2.6	1.8	3.1
	2.5 Σ + 0.7σ	8.9	10.4	11.9	10.8	6.8	11.8

## Discussion

This study is the first to report on the feasibility and accuracy of a carina-based registration for setup verification in esophageal cancer IGRT with the aid of fiducial markers. This was accomplished by comparing pCT-CBCT image registrations based on the bony anatomy (i.e., the current clinical standard) and on the carina. With the use of fiducial markers as a surrogate for tumor position, instead of delineated target volumes as used in previous studies, uncertainties from intra-observer delineation errors were avoided. Moreover, the use of fiducial markers allowed us to quantify the SE and sdREs in all directions, including the otherwise hard to determine CC direction [5, 17, 18]. Bony anatomy registration showed to be superior in terms of target coverage and demonstrated a smaller SE and, consequently, a smaller Σ and smaller required margins in most esophageal regions. Only for small tumors entirely confined to the mid-thoracic region, a small advantage in favor of a carina-based registration may exist.

The carina-based registration has been documented numerous times for IGRT in lung tumors [17, 19, 20]. First described in a series of 30 lung cancer patients, only including centrally located lesions, a high interobserver agreement with an excellent reproducibility was demonstrated using a carina-based registration, compared with a bony anatomy-based registration generating lower levels of reproducibility among observers [19]. However, in terms of target coverage, both registration methods provided complete coverage with no reported geographic misses, indicating that the carina or the bony anatomy could equally be used for accurate image registration in lung cancer patients [19]. Another series found a significant margin reduction in the CC direction from 1.24–0.82 cm using a carina-based registration for mediastinal lymph node irradiation in lung cancer IGRT [20]. A more recent study showed that, when a target volume consists of a lung volume and a mediastinal volume, a carina-based registration can be a superior setup verification method in terms of target coverage compared to the bony anatomy solely [17].

In contrast, for esophageal tumors, only one early study reported on carina-based registration in 20 patients [18]. This series compared different region of interest volumes to determine the optimal volume for an esophageal tumor registration; however, it suffered from several errors inherently connected with the use of a delineated target volume as a surrogate for tumor position and a cubic clipbox defined region of interest instead of a modern-shaped region of interest (i.e., a so-called mask) for registration [18]. In that series, smaller overall Σs in all directions were found; LR: 1.9 mm, CC: 2.3 mm, AP: 2.6 mm, when using a carina-based region of interest, compared to our Σs; LR: 3.3 mm, CC: 3.6 mm, and AP: 3.9 mm. This might be explained by the voluminous delineated structures, not taking deformation into account and making small differences undetectable. By calculating Pearson’s correlation coefficient, a poor correlation was found between the PTV-carina registration for lower located tumors and a good correlation for mid-thoracic tumors, which is similar to our findings (despite the fact that their findings were based on whole tumor volumes).

In the our series, an automatic carina-based registration was possible in 94.9% of registrations, in the remaining 5.1% a cumbersome manual registration was necessary making it prone to interobserver variation and human error. Further, this is considered impractical in the current clinical context. After registration, an adequate target volume coverage was reached in only 91.1% of the cases. Additionally, in two patients with distal tumors, an inadequate coverage was seen in over 1 of 3 registrations; presumably this is due to an unfavorable carina position for setup verification in esophageal cancer IGRT, because of patient-specific anatomy. This inadequate coverage might lead to a clinically relevant dosimetric impact on total dose; however, no conclusion can be drawn since this is beyond the scope of our investigation, but might be interesting for future research. However, with one registration approach showing 100% target coverage, one could argue the added value of such a comparison.

The only significant advantage of a bony anatomy-based registration was seen in the AP direction for distally located markers (Σ_carina_: 4.5 mm vs. Σ_bony_: 1.9 mm) (*p* = 0.004). This advantage might be due to the less concordant movement between esophagus and carina than initially expected, more pronounced in the distal region where a comparable rigidity in AP direction of the esophagus and the vertebrae is seen due to their similar anatomical borders. Moreover, enhancement of this discordance is imaginable due to the increased distance between the carina and lower esophagus [10, 18]. In the lower esophageal regions, gastric filling might introduce some interfractional position variation as well, this was recently investigated in a dosimetric study [21]. They stated that when an PTV margin of 1 cm is applied with a bony anatomy registration, dietary instructions do not contribute to optimal target coverage. CBCT before treatment does provide information on interfractional and interindividual variations in stomach volume for GEJ tumors but there is currently no need for adaptive treatment planning [21]. The SE and sdRE of interfractional marker position variation was expected to be affected by registration of the time-averaged CBCT with a snapshot pCT and possible artifacts (e.g., due to swallowing) or several anatomical changes during the radiation treatment course. However, since the same patients were used for both registrations, the SEs and sdREs were affected similarly, making comparison possible.

For our limited group of 24 patients, we calculated pro-forma margins, only including this interfractional position variation of the esophageal tumor (e.g., encompassing all four esophageal regions). Bony anatomy registration yielded overall margin contributions of 8.9 mm (LR), 11.9 mm (CC), and 6.8 mm (AP) (Table 2) [4]. The carina registrations yielded overall margin contributions of 10.4 mm (LR), 10.8 mm (CC), and 11.8 mm (AP), respectively. Thus, when using the carina instead of bony anatomy for patient setup verification, PTV volumes should be even larger. However, the margin estimate should be interpreted with caution due to the small sample size, inclusion of all four esophageal regions, the potential correlation between the marker position variations in one patient, and the exclusion of other errors (e.g., delineation, intrafractional) from the calculation. Only for mid-thoracic tumors was a required margin seen of only 8.0 mm vs. 8.9 mm in LR direction, 6.1 mm vs. 8.6 mm in CC direction, and 6.1 mm vs. 9.6 mm in AP direction, for a carina versus a bony registration respectively. Thus, it may be advocated to use a carina-based registration for tumors located in this mid-thoracic region. Nevertheless, target volumes in esophageal cancer typically extend across regions (including proximal and distal regions), making the net benefit of a carina registration clinically unfavorable compared with a bony anatomy registration.

The limitations of this study are the independent analysis of all markers separately, with no per patient/tumor correlation investigation being performed. A possible correlation between fiducial marker position variation and tumor/patients’ characteristics is possible, e.g., tumors in different regions, or patients with different features might show different movement properties. However, our sample size was not large enough to enable such a comparison. Another concern about carina registration is the possibility of motion artifacts resulting from the carina being captured during different positions of the respiratory cycle. This physiological motion on CBCT could cause a SE, as illustrated in a stereotactic liver study [22]. Using an averaged 4D-pCT for carina-based registration might result in smaller SE and sdRE.

Ideally, a direct daily marker-based registration would allow to account for interfractional tumor position variation. A previous study performed at our department, investigated and quantified the migration of these fiducial markers on the same data set as used in the current study [4]. They investigated the variation of pairwise distance between markers over the treatment course. Based on these results, they found some tissue deformation potentially induced by tumor regression, radiation toxicity, and/or different anatomical changes such as e.g., stomach filling over the treatment course, but with no evidence for marker migration. Nonetheless, because of this tissue deformation marker-based registration was shown to be infeasible. Even with deformable registration methods available, patient setup correction remains limited to a rigid transformation of the patient. This results in rigid bony anatomy registration still being the gold standard for routine daily clinical practice [4].

For future perspectives, the investigation of other registration volumes (e.g., PTV volume, diaphragm) and the individualization of image guidance methods on the basis of tumor location, may reduce residual error allowing the use of smaller margins. Additionally, the use of MRI guidance may allow direct tumor-based registration, reducing margins even further. At our institution, a bony anatomy-based registration with a visual marker assessment remains the optimal verification, ensuring that patient setup inaccuracies are corrected accurately and that any potential anatomical changes are identified and addressed on a day-to-day basis.

## Conclusions

A carina-based registration in comparison with a bony anatomy-based registration for esophageal cancer IGRT, results in inadequate target coverage in 8.9% of cases. Furthermore, large SEs and sdREs resulting in larger required anisotropic margins were seen after a carina-based registration. The carina-based registration might be slightly favorable only for tumors entirely confined to the mid-thoracic region. Therefore, our data endorse the use of bony-anatomy based registration over a carina-based registration for routine setup verification for esophageal tumor IGRT.

## Additional file

Additional file 1: **Figure** **S1.** Comparison of the distribution of the absolute mean systematic position errors (SE) of the individual markers relative to the carina (blue) and the bony anatomy (dark red). The distribution of absolute mean SE is given for each marker subgroup separately and compared between a carina-based registration and a bony anatomy-based registration. Results are given in the left-right, craniocaudal, and anterior-posterior direction. (EPS 113 kb)

## Abbreviations

3D: 3-dimensional
AP: Anterior-posterior
CBCT: Cone-beam CT
CC: Craniocaudal
CTV: Clinical target volume
eNAL: Extended no action level protocol
EUS: Endoscopic ultrasonography
GTV: Gross tumor volume
IGRT: Image-guided radiotherapy
kV/MV: Kilo−/megavoltage
LR: Left-right
M: Group mean: the mean of systematic error
pCT: Planning computed tomography
PTV: Planning target volume
SD: Standard deviation
sdRE: Random error
SE: Systematic error
XVI: X-ray volume imaging
Σ: Standard deviation of systematic error
σ: The root mean square of random error
